# E-Cigarette Use Among Adolescents Not Susceptible to Using Cigarettes

**DOI:** 10.5888/pcd15.170368

**Published:** 2018-02-01

**Authors:** Sarah D. Kowitt, Amira Osman, Leah M. Ranney, Courtney Heck, Adam O. Goldstein

**Affiliations:** 1 Department of Health Behavior, Gillings School of Global Public Health, University of North Carolina at Chapel Hill, Chapel Hill, North Carolina; 2 Lineberger Comprehensive Cancer Center, University of North Carolina at Chapel Hill, Chapel Hill, North Carolina; 3 Department of Family Medicine, University of North Carolina at Chapel Hill, Chapel Hill, North Carolina; 4 Division of Public Health, Tobacco Prevention and Control Branch, North Carolina Department of Health and Human Services, Raleigh, North Carolina

## Abstract

**Introduction:**

Research suggests that adolescents who use electronic cigarettes (e-cigarettes), including adolescents not susceptible to smoking cigarettes (ie, those who have never smoked cigarettes and are not attitudinally susceptible to using cigarettes), are more likely to initiate using cigarettes or other combustible tobacco products than adolescents who do not use e-cigarettes. In this study, we examined correlates of e-cigarette use and susceptibility among adolescents not susceptible to future cigarette smoking.

**Methods:**

We used data on high school students from the 2015 North Carolina Youth Tobacco Survey (n = 1,627). SAS logistic regression survey procedures were used to account for the complex survey design and sampling weights.

**Results:**

Increasing perceived harm of e-cigarettes was associated with lower odds of susceptibility to using e-cigarettes (adjusted odds ratio [AOR] = 0.79; 95% confidence interval [CI], 0.65–0.96) and current use of e-cigarettes (AOR = 0.43; 95% CI, 0.25–0.72). Similar patterns were found for perceived harm of secondhand e-cigarette vapor. Exposure to e-cigarette vapor in indoor or outdoor public places was positively associated with susceptibility to using e-cigarettes (AOR = 1.96; 95% CI, 1.33–2.91) and with current e-cigarette use (AOR = 5.69; 95% CI, 2.57–12.61).

**Conclusion:**

To prevent initiation of e-cigarette use, particularly among adolescents not susceptible to smoking cigarettes, educational campaigns could target harm perceptions associated with e-cigarettes. In addition, regulations that limit adolescents’ exposure to e-cigarettes in public places may decrease e-cigarette use by nonsusceptible adolescents.

## Introduction

The 2016 Surgeon General’s report labeled electronic cigarette (e-cigarette) use among adolescents and young adults a major public health concern (1); e-cigarettes were the most commonly used tobacco product among middle school students and high school students in 2015 (2). Several studies established the harmfulness of noncigarette tobacco products, including e-cigarettes, particularly for adolescents (3–6). These products contain nicotine, which harms the developing brains of adolescents (7). Moreover, early exposure to nicotine may lead to future tobacco product use (1).

E-cigarette use in adolescence is associated with future tobacco product use, including cigarettes (8–11). For example, in one study in California, high school students who reported using e-cigarettes at baseline were more likely to report use of cigarettes, cigars, and hookah during the next year than high school students who did not use e-cigarettes (8). Longitudinal research suggests that even adolescents not susceptible to smoking cigarettes (ie, adolescents who have never smoked cigarettes and are not attitudinally susceptible to using cigarettes) who use e-cigarettes are more likely to start smoking cigarettes in the future (8,12).

Accordingly, adolescents who are not susceptible to smoking cigarettes but who use e-cigarettes are a priority population for prevention of future tobacco product use. Despite the growing number of studies investigating e-cigarette use and susceptibility among adolescents and young adults (13–16), few studies examined differences in e-cigarette use and susceptibility among subgroups of adolescents (14). How are adolescents who are susceptible to using e-cigarettes different from adolescents who have tried or currently use e-cigarettes? The objective of this study was to examine correlates of e-cigarette use defined along a continuum (susceptibility to using e-cigarettes, ever use of e-cigarettes, and current use of e-cigarettes) among adolescents not susceptible to smoking cigarettes.

## Methods

We used data from the 2015 North Carolina Youth Tobacco Survey (NCYTS). The Tobacco Prevention and Control Branch of the North Carolina Department of Health and Human Services has administered the NCYTS every 2 years since 1999. Similar to the National Youth Tobacco Survey (17), the NCYTS is a public and charter school–based survey of students in grades 6 through 12 and is designed to provide data on the scope of the tobacco problem and progress toward overall state goals for reducing tobacco use among adolescents.

A multistage cluster sampling design was used. School districts were first selected within 3 geographic regions of the state. Classes were then randomly selected within each school. Participation was voluntary and anonymous. Passive consent forms were used, unless an active consent form was required according to a school district policy. The overall response rate was 74.4% for high school (grades 9 through 12) students (90.2% school response rate, 82.5% student response rate).

### Analytic sample

We restricted analyses to students in grades 9 through 12 who were not susceptible to smoking cigarettes. Previous research shows that susceptibility to smoking cigarettes predicts adolescents at risk for future smoking (18). We used 3 validated questions from Pierce et al (18) to assess susceptibility to cigarette smoking: 1) “Do you think you will smoke a cigarette in the next year?,” 2) “If one of your best friends were to offer you a cigarette, would you smoke it?,” and 3) “Do you think that you will try a cigarette soon?” For the first 2 questions, response options were “definitely yes,” “probably yes,” “probably not,” and “definitely not.” For the third question, response options were yes, no and “I have already tried smoking cigarettes.” We classified adolescents as not susceptible to smoking cigarettes (our analytic sample) as students who had never tried a cigarette and answered that they would “definitely not” smoke a cigarette in response to all 3 questions. A total of 1,681 high school students were not susceptible to smoking cigarettes. Of those, 54 participants (3.2%) had missing data on one or more study variables and were excluded from the analysis. Thus, our final analytic sample consisted of 1,627 high school students (Figure).

**Figure:**
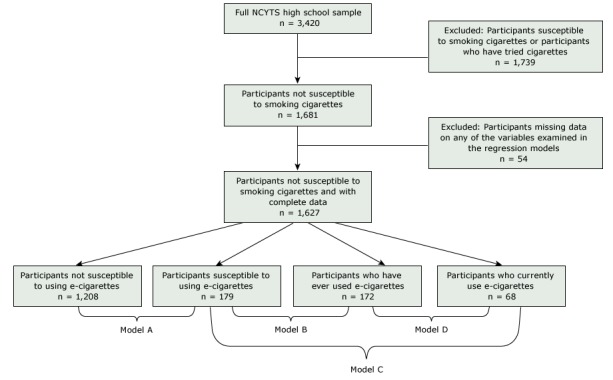
Flowchart of analytic sample for determining susceptibility to smoking cigarettes, NCYTS, 2015. Three validated questions from Pierce et al (18) were used to assess susceptibility to smoking cigarettes. Abbreviation: NCYTS, North Carolina Youth Tobacco Survey.

### Measures

#### Progression of e-cigarette use 

We defined our dependent variable, progression of e-cigarette use, as one of 4 categories along a continuum: 1) a current e-cigarette user, 2) an ever e-cigarette user, 3) a never e-cigarette user susceptible to e-cigarette use, and 4) a never e-cigarette user not susceptible to e-cigarette use.

We used 2 items to determine current and ever use of e-cigarettes: 1) “Have you ever used an e-cigarette, even one or two puffs?” (with response options of yes and no) and 2) “During the past 30 days, on how many days did you use an e-cigarette” (with response options of 0 days, 1 or 2 days, 3 to 5 days, 6 to 9 days, 10 to 19 days, 20 to 29 days, and all 30 days). We defined adolescents as current e-cigarette users if they reported ever using an e-cigarette and reported using an e-cigarette on at least one of the past 30 days. We defined adolescents as ever e-cigarette users if they reported ever using an e-cigarette but not in the past 30 days.

Although no uniformly agreed upon measure of susceptibility to using e-cigarettes exists, researchers have used various measures, including some or all of Pierce et al’s measure of susceptibility to smoking cigarettes (18) (replacing “cigarettes” with “e-cigarettes”) alone (15) or in combination with other items (16). We used 2 items from Pierce et al’s measure of susceptibility to cigarettes to determine susceptibility to using e-cigarettes: 1) “Do you think you will try an e-cigarette in the next year?” and 2) “If one of your best friends were to offer you an e-cigarette, would you use it?” For both items, response options were “definitely yes,” “probably yes,” “probably not,” and “definitely not.” Only students who had never used an e-cigarette and answered that they would “definitely not” use an e-cigarette in response to both questions were classified as not susceptible to using e-cigarettes. Otherwise, they were classified as susceptible to using e-cigarettes.

#### Perceptions and exposures

The survey assessed perceived harm of e-cigarette use by asking participants, “How harmful are electronic cigarettes to your health?” Response options were provided on a 4-point scale: 1, not sure or not at all harmful; 2, somewhat harmful; 3, very harmful; and 4, extremely harmful.

The survey assessed perceived harm of secondhand e-cigarette vapor by asking participants, “Do you think that breathing vapor from other people’s e-cigarettes is . . . ?” Response options were provided on a 4-point scale: 1, not harmful at all to one’s health; 2, not very harmful to one’s health; 3, somewhat harmful to one’s health, or 4, very harmful to one’s health.

We assessed exposure to e-cigarette use in public places and at home with 2 questions, asking participants 1) whether they had been exposed to vapor from e-cigarettes in the past 7 days in an indoor or outdoor public place and 2) whether someone who lives with them now uses e-cigarettes. We classified participants as exposed to e-cigarette vapor in a public place if they reported breathing e-cigarette vapor on 1 or more days or, if otherwise, not exposed. Similarly, we classified participants as exposed to e-cigarettes at home if they reported living with someone who uses e-cigarettes or, if otherwise, not exposed.

We assessed the exposure to online tobacco advertising (including e-cigarettes) on the Internet by asking participants, “When you are using the Internet, how often do you see ads for tobacco products, including electronic cigarettes or e-cigarettes?” Response options were provided on a 5-point scale: 1, never or “I do not use the Internet”; 2, rarely; 3, sometimes; 4, most of the time; and 5, always.

#### Demographics

Demographic variables included sex (female or male), age (treated as a continuous variable), and race/ethnicity, categorized as non-Hispanic white, non-Hispanic black, Hispanic, or non-Hispanic other race.

### Statistical analysis

We used χ^2^ tests and analysis of variance (ANOVA) tests to test bivariate associations among independent variables and e-cigarette use. We then conducted 4 multivariate logistic regressions, estimating the odds of being susceptible to using e-cigarettes versus not being susceptible (the referent group) (model A), the odds of being an ever e-cigarette user versus being susceptible to using e-cigarettes (the referent group) (model B), the odds of being a current e-cigarette user versus being susceptible to using e-cigarettes (the referent group) (model C), and the odds of being a current e-cigarette user versus ever using e-cigarettes (the referent group) (model D). We tabulated weighted percentages, adjusted odds ratios (AORs), and 95% confidence intervals (CIs). Analyses used SAS version 9.4 survey procedures (SAS Institute Inc). We set an α of .05 and used 2-tailed tests.

## Results

In our sample of adolescents not susceptible to smoking cigarettes (n = 1,627), half identified as non-Hispanic white (50.9%) and more than one-quarter identified as non-Hispanic black (31.1%) (Table 1). Twenty-six percent of these adolescents were at high risk for future e-cigarette use: 11.3% were classified as susceptible to using e-cigarettes, 10.4% were ever e-cigarette users; and 4.5% were current e-cigarette users. On average, participants reported e-cigarettes to be somewhat harmful to one’s health (mean rating, 2.1; 95% CI, 2.1–2.2) and e-cigarette vapor to be somewhat harmful to one’s health (mean rating, 2.9; 95% CI, 2.8–3.0).

**Table 1:** Sample Characteristics of High School Students Not Susceptible to Smoking Cigarettes (n = 1,627), North Carolina Youth Tobacco Survey, 2015

Variable	No.	%^a^ or Mean (95% Confidence Interval)
**Sex**		
Female	818	50.7 (47.3–54.1)
Male	809	49.3 (45.9–52.7)
**Age, y**	1,627	15.6 (15.4–15.7)
**Race/ethnicity**		
Non-Hispanic white	899	50.9 (46.0–55.8)
Non-Hispanic black	429	31.1 (26.7–35.5)
Hispanic	208	10.6 (8.5–12.8)
Non-Hispanic other	91	7.4 (4.5–10.3)
**Susceptibility to e-cigarette use**		
Not susceptible to using e-cigarettes	1,208	73.9 (70.9–76.9)
Susceptible to using e-cigarettes	179	11.3 (9.5–13.0)
Ever e-cigarette user	172	10.4 (8.6–12.1)
Current e-cigarette user	68	4.5 (3.2–5.7)
**Exposure to e-cigarette vapor in indoor or outdoor public places in the past 7 days**		
No	1,338	83.4 (81.1–85.7)
Yes	289	16.6 (14.3–18.9)
**Exposure to e-cigarettes at home**		
No	1,546	94.9 (94.0–95.9)
Yes	81	5.1 (4.1–6.0)
**Exposure to online tobacco advertising (including e-cigarettes), mean rating^b^**	1,627	2.5 (2.4–2.5)
**Perceived harm, mean rating**		
Of e-cigarettes^c^	1,627	2.1 (2.1–2.2)
Of secondhand e-cigarette vapor^d^	1,627	2.9 (2.8–3.0)

Abbreviation: e-cigarette, electronic cigarette.

a Percentages are weighted estimates.

b Rated on a 5-point scale. Participants were asked, “When you are using the Internet, how often do you see ads for tobacco products, including electronic cigarettes or e-cigarettes?” Response options were 1, never or “I do not use the Internet”; 2, rarely; 3, sometimes; 4, most of the time; and 5, always.

c Rated on a 4-point scale. Participants were asked, “How harmful are electronic cigarettes to your health?” Response options were 1, not sure or not at all harmful; 2, somewhat harmful; 3, very harmful; and 4, extremely harmful.

d Rated on a 4-point scale. Participants were asked, “Do you think that breathing vapor from other people’s e-cigarettes is . . . ?” Response options were 1, not harmful at all to one’s health; 2, not very harmful to one’s health; 3, somewhat harmful to one’s health; or 4, very harmful to one’s health.

In bivariate analyses, several variables were significantly associated with e-cigarette susceptibility and use among adolescents not susceptible to smoking cigarettes (Table 2). Older adolescents were more likely to be ever or current e-cigarette users (*P* < .001). A greater proportion of current e-cigarette users (62.9%), ever e-cigarette users (21.4%), and adolescents susceptible to using e-cigarettes (22.0%) reported being exposed to e-cigarette vapor in public places, compared with adolescents not susceptible to using e-cigarettes (12.3%) (*P* < .001). Similarly, a greater proportion of current e-cigarette users (14.5%), ever e-cigarette users (11.4%), or adolescents susceptible to using e-cigarettes (6.5%) reported living with someone who now uses e-cigarettes, compared with adolescents not susceptible to using e-cigarettes (3.4%) (*P* < .001). Finally, current e-cigarette users, ever e-cigarette users, and adolescents susceptible to using e-cigarettes perceived e-cigarettes and secondhand e-cigarette vapor to be less harmful than adolescents not susceptible to using e-cigarettes (*P* < .001).

**Table 2:** Bivariate Associations, Stratified by Category of E-Cigarette Susceptibility and Use, Among High School Students Not Susceptible to Smoking Cigarettes, Weighted Estimates, North Carolina Youth Tobacco Survey, 2015^a^

Variable	Not Susceptible to Using E-Cigarettes (n = 1,208)	Susceptible to Using E-Cigarettes (n = 179)	Ever E-Cigarette User (n = 172)	Current E-Cigarette User (n = 68)	*P* Value^b^
**Sex**					
Female	610 (51.1)	91 (49.3)	84 (47.8)	33 (54.3)	.82
Male	598 (48.9)	88 (50.7)	88 (52.2)	35 (45.7)	
**Age, mean (SE), y**	15.5 (0.1)	15.5 (0.1)	15.9 (0.1)	16.1 (0.1)	<.001
**Race/ethnicity**					
Non-Hispanic white	666 (50.7)	101 (51.7)	96 (52.8)	36 (47.6)	.03
Non-Hispanic black	302 (28.9)	49 (35.8)	56 (37.8)	22 (38.6)	
Hispanic	161 (11.4)	24 (10.2)	15 (6.9)	8 (7.5)	
Non-Hispanic other	79 (8.8)	5 (2.3)	5 (2.5)	2 (6.3)	
**Exposure to e-cigarette vapor in indoor or outdoor public places in the past 7 days**					
No	1,043 (87.7)	135 (78.0)	133 (78.6)	27 (37.1)	<.001
Yes	165 (12.3)	44 (22.0)	39 (21.4)	41 (62.9)	
**Exposure to e-cigarettes at home**					
No	1,165 (96.6)	167 (93.5)	153 (88.6)	61 (85.5)	<.001
Yes	43 (3.4)	12 (6.5)	19 (11.4)	7 (14.5)	
**Exposure to online tobacco advertising (including e-cigarettes),^c^ mean rating (SE)**	2.5 (0.04)	2.4 (0.08)	2.5 (0.08)	2.7 (0.1)	.17
**Perceived harm, mean rating (SE)**					
Of e-cigarettes^d^	2.3 (0.03)	1.9 (0.08)	1.8 (0.07)	1.5 (0.05)	<.001
Of secondhand e-cigarette vapor^e^	3.1 (0.03)	2.7 (0.07)	2.5 (0.07)	2.0 (0.09)	<.001

Abbreviation: e-cigarette, electronic cigarette.

a Values are number (weighted percentage) unless otherwise indicated. Data are from 1,627 high school students not susceptible to smoking cigarettes.

b χ^2^ tests for categorical variables and analysis of variance (ANOVA) tests for continuous variables.

c Rated on a 5-point scale. Participants were asked, “When you are using the Internet, how often do you see ads for tobacco products, including electronic cigarettes or e-cigarettes?” Response options were 1, never or “I do not use the Internet”; 2, rarely; 3, sometimes; 4, most of the time; and 5, always.

d Rated on a 4-point scale. Participants were asked, “How harmful are electronic cigarettes to your health?” Response options were 1, not sure or not at all harmful; 2, somewhat harmful; 3, very harmful; and 4, extremely harmful.

e Rated on a 4-point scale. Participants were asked, “Do you think that breathing vapor from other people’s e-cigarettes is . . . ?” Response options were 1, not harmful at all to one’s health; 2, not very harmful to one’s health; 3, somewhat harmful to one’s health; or 4, very harmful to one’s health.

### Model A: odds of being susceptible to using e-cigarettes versus not being susceptible

Non-Hispanic adolescents of other races had lower odds than non-Hispanic white adolescents of being susceptible to using e-cigarettes (AOR = 0.33; 95% CI, 0.11–0.97) (Table 3). In addition, adolescents exposed to e-cigarette vapor in public places, compared with adolescents not exposed, had higher odds of being susceptible to using e-cigarettes (AOR = 1.96; 95% CI, 1.33–2.91). Finally, as perceived harm of e-cigarettes (AOR = 0.79; 95% CI, 0.65–0.96) and secondhand e-cigarette vapor (AOR = 0.73; 95% CI, 0.62–0.86) increased, adolescents had lower odds of being susceptible to using e-cigarettes.

**Table 3:** Multivariable Logistic Regressions for E-Cigarette Use Among High School Students Not Susceptible to Smoking Cigarettes, Weighted Estimates, North Carolina Youth Tobacco Survey, 2015^a^

Variable	Model A: Susceptible to Using E-Cigarettes (n = 179) vs Not Susceptible (n = 1,208)	Model B: Ever E-Cigarette Use (n = 172) vs Susceptible to Using e-Cigarettes (n = 179)	Model C: Current E-Cigarette Use (n = 68) vs Susceptible to Using e-Cigarettes (n = 179)	Model D: Current E-Cigarette Use (n = 68) vs Ever Use (n = 172)
**Sex**				
Female	1 [Reference]	1 [Reference]	1 [Reference]	1 [Reference]
Male	0.94 (0.64–1.36)	1.12 (0.63–1.99)	0.61 (0.22–1.72)	0.71 (0.31–1.63)
**Age**	1.00 (0.84–1.19)	1.31 (1.03–1.67)^b^	1.99 (1.41–2.81)^b^	1.32 (1.04–1.69)^b^
**Race/ethnicity**				
Non-Hispanic white	1 [Reference]	1 [Reference]	1 [Reference]	1 [Reference]
Non-Hispanic black	1.42 (0.87–2.33)	1.13 (0.61–2.09)	2.29 (0.91–5.77)	1.48 (0.64–3.43)
Hispanic	1.03 (0.58–1.85)	0.77 (0.34–1.72)	1.68 (0.48–5.93)	1.49 (0.54–4.12)
Non-Hispanic other	0.33 (0.11–0.97)^b^	1.13 (0.33–3.84)	5.55 (1.41–21.91)^b^	2.62 (0.52–13.08)
**Exposure to e-cigarette vapor in indoor or outdoor public places in the past 7 days**				
No	1 [Reference]	1 [Reference]	1 [Reference]	1 [Reference]
Yes	1.96 (1.33–2.91)^b^	0.78 (0.43–1.42)	7.82 (3.35–18.27)^b^	5.69 (2.57–12.61)^b^
**Exposure to e-cigarettes at home**				
No	1 [Reference]	1 [Reference]	1 [Reference]	1 [Reference]
Yes	1.55 (0.79–3.07)	1.99 (0.85–4.64)	2.59 (0.80–8.36)	0.93 (0.18–4.71)
**Exposure to online tobacco advertising (including e-cigarettes)^c^**	0.84 (0.65–1.09)	1.24 (0.91–1.70)	1.29 (0.82–2.04)	1.17 (0.76–1.78)
**Perceived harm**				
Of e-cigarettes^d^	0.79 (0.65–0.96)^b^	0.85 (0.65–1.10)	0.31 (0.14–0.66)^b^	0.43 (0.25–0.72)^b^
Of secondhand e-cigarette vapor^e^	0.73 (0.62–0.86)^b^	0.79 (0.63–0.98)^b^	0.46 (0.29–0.73)^b^	0.71 (0.53–0.94)^b^

Abbreviation: e-cigarette, electronic cigarette.

a Data are from 1,627 high school students not susceptible to smoking cigarettes.

b Significant at *P* < .05.

c Rated on a 5-point scale. Participants were asked, “When you are using the Internet, how often do you see ads for tobacco products, including electronic cigarettes or e-cigarettes?” Response options were on a 5-point scale: 1, never or “I do not use the Internet”; 2, rarely; 3, sometimes; 4, most of the time; and 5, always.

d Rated on a 4-point scale. Participants were asked, “How harmful are electronic cigarettes to your health?” Response options were 1, not sure or not at all harmful; 2, somewhat harmful; 3, very harmful; and 4, extremely harmful.

e Rated on a 4-point scale. Participants were asked, “Do you think that breathing vapor from other people’s e-cigarettes is . . . ?” Response options were 1, not harmful at all to one’s health; 2, not very harmful to one’s health; 3, somewhat harmful to one’s health; or 4, very harmful to one’s health.

### Model B: odds of being an ever e-cigarette user versus being susceptible to using e-cigarettes

Except for age and perceived harm of secondhand e-cigarette vapor, we found no significant differences for any of the study variables between susceptible adolescents and those who ever used an e-cigarette. Older age was associated with higher odds of ever having used an e-cigarette (AOR = 1.31; 95% CI, 1.03–1.67). In addition, greater perceived harm of secondhand e-cigarette vapor was associated with lower odds of being an ever user of e-cigarettes (AOR = 0.79; 95% CI, 0.63–0.98).

### Model C: odds of being a current e-cigarette user versus being susceptible to using e-cigarettes

As age increased, adolescents had higher odds of being a current user of e-cigarettes than of being susceptible to using e-cigarettes (AOR = 1.99; 95% CI, 1.41–2.81). In addition, adolescents exposed to e-cigarette vapor in public places, compared to those not exposed, had higher odds of being a current user of e-cigarettes (AOR = 5.55; 95% CI, 1.41–21.91). Finally, greater perceived harm of e-cigarettes (AOR = 0.31; 95% CI, 0.14–0.66) and of secondhand e-cigarette vapor (AOR = 0.46; 95% CI, 0.29–0.73) were associated with lower odds of being a current user of e-cigarettes.

### Model D: odds of being a current e-cigarette user versus being an ever user

As age increased, adolescents had higher odds of being a current user of e-cigarettes than of being an ever user (AOR = 1.32; 95% CI, 1.04–1.69). In addition, adolescents exposed to e-cigarette vapor in public places, compared to those not exposed, had higher odds of being a current user of e-cigarettes than of being an ever user (AOR = 5.69; 95% CI, 2.57–12.61). Finally, greater perceived harm of e-cigarettes (AOR = 0.43; 95% CI, 0.25–0.72) and of secondhand e-cigarette vapor (AOR = 0.71; 95% CI, 0.53–0.94) were associated with lower odds of being a current user of e-cigarettes.

## Discussion

To our knowledge, our study is the first to analyze correlates of e-cigarette use among adolescents not susceptible to smoking cigarettes. Our study identified several modifiable factors associated with e-cigarette susceptibility and use among this priority population. We found that adolescents who thought that e-cigarettes and secondhand e-cigarette vapor were not harmful or somewhat harmful had higher odds of being susceptible to using e-cigarettes, ever using e-cigarettes, or currently using e-cigarettes than adolescents who thought e-cigarettes and secondhand e-cigarette vapor were harmful. Moreover, we found that exposure to e-cigarette vapor in indoor or outdoor public places was associated with greater odds of being susceptible to using e-cigarettes or currently using e-cigarettes. This research is important for public health efforts to decrease adolescent tobacco use in several ways.

First, that adolescents who are otherwise not susceptible to smoking cigarettes may use e-cigarettes is a major public health problem. Using school enrollment figures for North Carolina (454,963 high school students), we estimated that 55,725 high school students in North Carolina are at low risk of smoking cigarettes (ie, not susceptible) but at high risk for sustained e-cigarette use (ie, they are susceptible to using e-cigarettes, have experimented with e-cigarettes, or currently use e-cigarettes). On a national scale, these findings would apply to millions of adolescents, which is particularly worrisome given the harmfulness of nicotine to adolescents’ brain development (7).

Second, adolescents not susceptible to smoking cigarettes but who use e-cigarettes are clearly at an increased risk for nicotine addiction and future cigarette and tobacco use (8,12). From previous research, we know that adolescents susceptible to smoking cigarettes are more likely to believe e-cigarettes are less harmful than cigarettes (19) and are more likely to be past or current e-cigarette users than are adolescents not susceptible to smoking cigarettes (20). However, no studies, to our knowledge, have focused on adolescents not susceptible to smoking cigarettes. Given associations between age and e-cigarette use in our study and others (21), tobacco education programs and media campaigns tailored to this population could prevent future generations of tobacco users and focus on younger or older audiences, depending on whether the goal is to prevent initiation, increase cessation, or decrease experimentation. Moreover, educational strategies focused on educating adolescents about the risks of e-cigarettes may be particularly useful (22), given associations between perceived harm of e-cigarettes and susceptibility in our study and prior research (23). To avoid unintended consequences, educational programs will also need to emphasize that e-cigarettes are not safe but are also likely not as harmful as combustible cigarettes. California became one of the first states to feature a campaign on the dangers of e-cigarettes with television and digital advertisements (24).

Third, we found that adolescents exposed to e-cigarette vapor in indoor or outdoor public places had higher odds of being susceptible to using e-cigarettes or currently using e-cigarettes than adolescents not exposed. These findings suggest that local or state regulations controlling secondhand exposure to vapor of e-cigarettes in public places (eg, school buildings, stores, restaurants, school grounds, parks) will be a useful strategy. Multiple states, localities, and health organizations have already banned use of these products in public settings where cigarette smoking is prohibited (25), including hospitals (26). However, other states have drafted laws in which e-cigarettes are excluded from the definitions of tobacco products or smoking, which could undermine regulation (27). Moving forward, clean air regulations that include e-cigarettes at local, state, and federal levels will be important in decreasing initiation, experimentation, and exposure to tobacco products, including e-cigarettes.

Finally, in contrast to previous research (28), we found no association between self-reported exposure to Internet advertisements about tobacco products (including e-cigarettes) and e-cigarette use among adolescents not susceptible to smoking cigarettes. It is possible that our measure of exposure to online tobacco advertising, including advertising for e-cigarettes (which was phrased as “When you are using the Internet, how often do you see ads for tobacco products, including electronic cigarettes or e-cigarettes?”) was not specific enough to capture data on exposure to e-cigarette advertisements only. We also found no association between exposure to e-cigarettes at home and e-cigarette susceptibility or use. Future research, using more exact measures, should examine these and other factors (eg, curiosity [29], peer influence [30], different forms of marketing [15]) and their influence on e-cigarette use in this population.

We acknowledge several limitations. First, our data are cross-sectional, which makes it difficult to determine temporality of associations and precludes determination of causality. Second, our findings may not be generalizable to other states or locations in the United States. Third, no standard measure of susceptibility to using e-cigarettes exists. Although our measure was informed by previous research on susceptibility to cigarettes (18), future studies may determine more nuanced ways of measuring e-cigarette susceptibility. Finally, data used in this study were self-reported by adolescents and are subject to related biases such as social desirability. As such, adolescents may have overreported or underreported their smoking behaviors. Despite these limitations, our study provides new information on correlates of e-cigarette use and susceptibility in a large sample of adolescents.

The 2016 Surgeon General’s report called for a multipronged approach to address e-cigarette use (1). Our study provides necessary evidence on correlates of e-cigarette use and susceptibility and some of the first data on a unique population of adolescents at low risk for cigarette use. Our findings of associations between perceived harm of e-cigarettes, exposure to e-cigarette vapor in public places, and e-cigarette susceptibility and use may aid future public health interventions to reduce e-cigarette use among adolescents and young adults.
